# Age-associated changes in the impact of sex steroids on influenza vaccine responses in males and females

**DOI:** 10.1038/s41541-019-0124-6

**Published:** 2019-07-12

**Authors:** Tanvi Potluri, Ashley L. Fink, Kristyn E. Sylvia, Santosh Dhakal, Meghan S. Vermillion, Landon vom Steeg, Sharvari Deshpande, Harish Narasimhan, Sabra L. Klein

**Affiliations:** 1 W. Harry Feinstone Department of Molecular Microbiology and Immunology, Johns Hopkins University Bloomberg School of Public Health, Baltimore, MD 21205 USA; 2 Department of Molecular and Comparative Pathobiology, The Johns Hopkins School of Medicine, Baltimore, MD 21201 USA; 3 Department of Biochemistry and Molecular Biology, Johns Hopkins University Bloomberg School of Public Health, Baltimore, MD 21205 USA; 4 Present Address: Biology Department, College of Saint Benedict and Saint John’s University, Collegeville, MN 56321 USA

**Keywords:** Humoral immunity, Influenza virus

## Abstract

Vaccine-induced immunity declines with age, which may differ between males and females. Using human sera collected before and 21 days after receipt of the monovalent A/Cal/09 H1N1 vaccine, we evaluated cytokine and antibody responses in adult (18–45 years) and aged (65+ years) individuals. After vaccination, adult females developed greater IL-6 and antibody responses than either adult males or aged females, with female antibody responses being positively associated with concentrations of estradiol. To test whether protection against influenza virus challenge was greater in females than males, we primed and boosted adult (8–10 weeks) and aged (68–70 weeks) male and female mice with an inactivated A/Cal/09 H1N1 vaccine or no vaccine and challenged with a drift variant A/Cal/09 virus. As compared with unvaccinated mice, vaccinated adult, but not aged, mice experienced less morbidity and better pulmonary viral clearance following challenge, regardless of sex. Vaccinated adult female mice developed antibody responses that were of greater quantity and quality and more protective than vaccinated adult males. Sex differences in vaccine efficacy diminished with age in mice. To determine the role of sex steroids in vaccine-induced immune responses, adult mice were gonadectomized and hormones (estradiol in females and testosterone in males) were replaced in subsets of animals before vaccination. Vaccine-induced antibody responses were increased in females by estradiol and decreased in males by testosterone. The benefit of elevated estradiol on antibody responses and protection against influenza in females is diminished with age in both mice and humans.

## Influenza vaccines: Sex differences in responses lost with age

Males and females show pronounced differences in their immune responses. Sabra L. Klein and colleagues at Johns Hopkins University examine how sex differences in immune responses to influenza vaccination are affected by aging. Using both human volunteers and mouse models (adults vs. aged) they find that adult females have more robust interleukin-6 production and greater titers and quality of influenza-specific antibodies. Vaccination was also more protective in adult female mice. These sex differences in influenza responses are however lost in aged individuals. Gonadectomy and hormone replacement demonstrate that sex steroids exert a key influence, with estradiol positively- and testosterone negatively-correlating with influenza vaccine responses. The diminished production of estradiol in aged females and testosterone in aged males likely contributes to the equalization of influenza vaccine responses in the aged.

## Introduction

Aging is associated with a progressive decline and remodeling of the immune system that leaves aged individuals (i.e., people 65 years and older) at an increased risk of severe outcome from infectious diseases, including influenza.^1^ For vaccine preventable diseases, including influenza, aged individuals are labeled “an at-risk population”, and are targeted for vaccine campaigns.^2^ Although the effects of age on the adaptive immune response to vaccines are well documented,^3^ the extent to which the sexes differ in their immune responses to vaccines over the life course, including in older age, remains relatively understudied.

In general, adaptive immune responses to vaccines are lower in both aged males and females as compared with their younger adult counterparts.^4^ While it is presumed that males and females experience the same overall immune-related changes with age, males tend to experience a more dramatic decrease in total T and B cell populations and an increase in senescent CD8+ T cells as compared to females.^5–8^ In contrast, aged females produce greater numbers of age-associated B cells (ABC), a functionally unique B cell subset that is associated with anti-viral antibody production, as compared to aged males.^9,10^

Sex (i.e., biological construct defined by sex chromosomes, reproductive tissues, and sex hormones) and gender (i.e., social construct, including identification through self-reporting) differences in antibody responses to vaccines administered to aged individuals have been reported in a few studies, but vary depending on the vaccine antigen. For example, aged females reportedly have greater hemagglutinin inhibition (HAI) antibody responses to both seasonal and pandemic influenza vaccines than males, whereas aged males appear to have greater antibody responses to the pneumococcal and tetanus, diphtheria, and pertussis vaccines than females.^4^ The lack of consistency in the sex-specific vaccine-induced antibody responses may reflect the limited number of studies that have partitioned and analyzed data for sex-related differences, the vaccine formulations and dosages, or even the biological and social differences between the sexes.^11^

Influenza vaccines are recommended for all individuals aged 6 months and older and are administered annually. Sex/gender differences in response to influenza vaccines in both adult and aged individuals have been reported.^12^ Data from human trials have shown that when adults, ages 18–49 years, are administered either a full dose or half dose of the seasonal trivalent inactivated influenza vaccine (TIV), females generate HAI antibody titers that are twice as high as those of males.^13^ Similarly, adult females 20–89 years of age (not partitioned by age or reproductive status) generate greater neutralizing antibody titers to the H3N2 and influenza B antigens following seasonal TIV than males, and males who have the highest circulating testosterone concentrations tend to have the lowest neutralizing antibody titers,^14^ but this effect has been challenged.^15^ In response to the pandemic monovalent 2009 H1N1 vaccine administered to older individuals (ages 61–86 years), aged females generate greater HAI antibody titers than males, which results in a 2–3 times higher seroconversion rate for females as compared to males.^16^ It also has been demonstrated, at least in one study, that among community-dwelling older individuals in Taiwan who received the seasonal influenza vaccine, higher HAI titers were associated with lower hospitalization rates and mortality in females as compared to males.^17^ These data suggest that influenza vaccine efficacy may be greater for aged females as compared to aged males. Overall, there are no clinical studies that have adequately partitioned and analyzed data for age-related sex differences in the context of reproductive status.

Animal models provide further evidence for sex differences in the immunological responses to and protection provided from influenza vaccines. Following vaccination with either inactivated influenza vaccine or TIV, adult female mice generate greater influenza-specific antibody responses and are better protected following challenge than adult male mice.^18–20^ In response to live influenza virus infection, adult female mice also exhibit greater innate immune responses, have higher influenza-specific antibody responses, have more virus-specific resident memory T cells in their lungs, and are better protected from secondary heterosubtypic challenge than male mice.^19,21,22^ Because our lab and others have shown that adult female mice exhibit heightened immune responses to influenza infection and vaccination, in the current study, we sought to evaluate how sex/gender, age, and sex steroid hormones impact the humoral immune response following receipt of an inactivated monovalent 2009 H1N1 vaccine in humans and mice. Further, we aimed to use our mouse model to evaluate the impact of sex and age on vaccine efficacy (i.e., protection following challenge).

## Results

### In humans, females have higher antibody titers post vaccination among adult, but not aged, vaccinees

Seasonal influenza vaccination induces proinflammatory responses immediately following receipt of TIV, which can be dependent on both the reported age and gender (i.e., self-reporting of being male or female) of the vaccinee.^14^ To test the hypothesis that gender- and age-associated differences in proinflammatory immune responses occur immediately following receipt of the monovalent 2009 H1N1 vaccine, serum concentrations of 10 cytokines were measured prior to and 21 days post-vaccination in samples collected from adult (18–45 years [i.e., reproductive], *n* = 20 males and 30 females) and aged (65+ years [i.e., non-reproductive], *n* = 47 females and 48 males) individuals. Only IFNγ, IL-6, Il-8, IL-10, and TNFα were detected in serum and increased following vaccination (*p* < 0.05 in each case, one-way ANOVAs). Neither gender nor age influenced serum concentrations of IFNγ, TNFα, IL-10, or IL-8; young adult females, however, had higher concentrations of IL-6 post-vaccination than either young adult males or aged adult females (Table 1, *p* *=* 0.06, one-way ANOVA). Because IL-6 is crucial for germinal center formation, antibody production, and class switching,^23^ elevated IL-6 concentrations immediately after vaccination might suggest that young adult females should also have higher vaccine-induced antibody responses than either young adult males or aged adult females.

**Table 1:** Mean fold change ± standard error of the mean of cytokines in male and female human serum samples

Cytokine	Adult		Aged	
	Male	Female	Male	Female
TNFα	1.03 ± 0.03	1.19 ± 0.09	1.08 ± 0.13	1.03 ± 0.11
IFNɣ	0.94 ± 0.08	1.05 ± 0.11	1.02 ± 0.08	1.05 ± 0.11
IL-6	0.79 ± 0.13	2.10 ± 0.87*	1.17 ± 0.29	1.32 ± 2.03
IL-8	0.94 ± 0.08	1.05 ± 0.11	1.02 ± 0.08	1.07 ± 0.10
IL-10	1.14 ± 0.14	2.32 ± 0.57	2.11 ± 1.29	2.39 ± 0.60

*Difference between adult males and females, *p* = 0.06

**Fig. 1:**
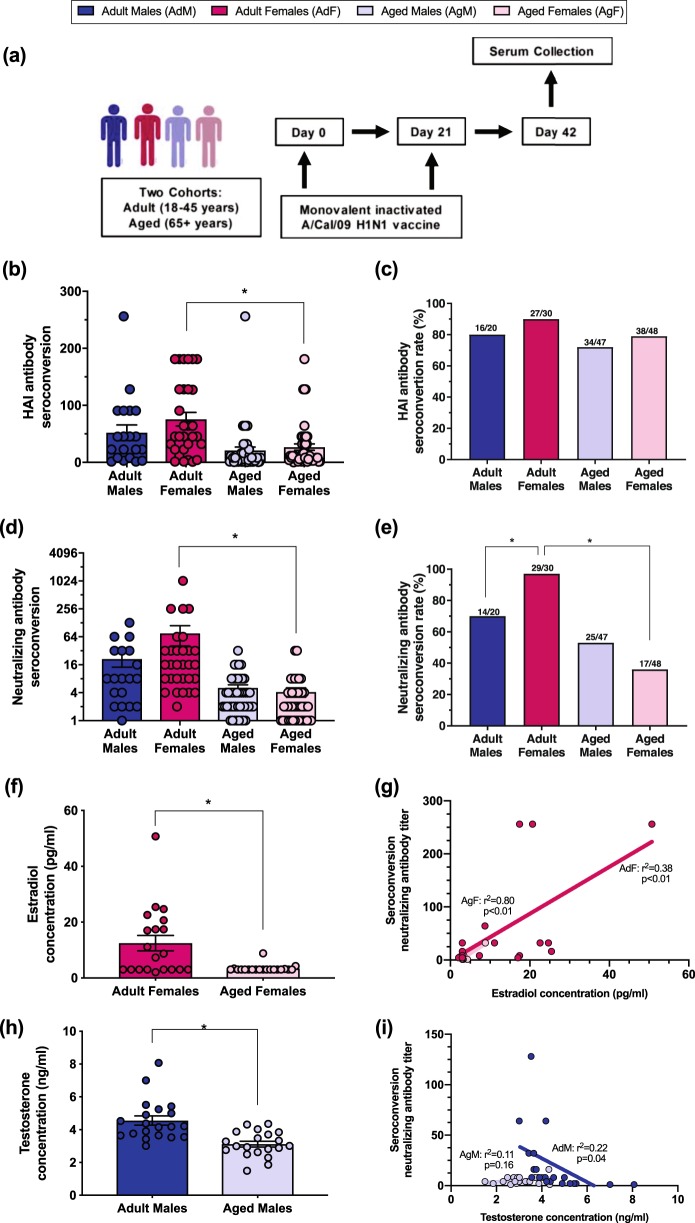
Adult females of reproductive ages have greater seroconversion to a monovalent A/Cal/09 H1N1 vaccine, which is associated with concentrations of estradiol. Adult (18–45 years) and aged (65+ years) males (dark or light blue, respectively) and females (dark or light pink, respectively) were vaccinated with an inactivated, monovalent 2009 H1N1 vaccine and serum was collected prior to and at day 42 (i.e., 21 days after boost) (**a**). Seroconversion of hemagglutination inhibition titers after vaccination (**b**) and the proportion of individuals with ≥4-fold increase in HAI antibody titers after vaccination (i.e., rate of seroconversion) (**c**) were measured. Seroconversion of neutralizing antibody titers after vaccination (**d**) and the rate of seroconversion after vaccination (**e**) was measured. Serum concentrations of estradiol (E2) and testosterone (T) were measured (**f**, **h**) and the correlation between hormone concentrations and neutralizing antibody seroconversion was quantified using a linear regression model (**g**, **i**). Data represent mean ± standard error of the mean and significant differences between groups are denoted by asterisks (*p < 0.05) based on one-way ANOVAs (**b**, **d**), Chi square analyses (**c**, **e**), or *t*-tests (**f**, **h**). *n* = 20 adult males, 30 adult females, 47 aged males, and 48 aged females

To date, no published human studies have partitioned and analyzed gender- and age-associated differences in antibody responses to influenza vaccination based on reproductive status. To test the hypothesis that male–female differences in vaccine-induced immunity are dependent on age/reproductive status, we compared serum antibody responses among adult and aged individuals following vaccination with a monovalent 2009 H1N1 vaccine (Fig. 1a). In humans, antibody titers are traditionally measured with the HAI assay that tests the ability of antibody to prevent agglutination of red blood cells by influenza virus.^24^ Similar to some previous studies that reported no differences between males and females in HAI titers following receipt of seasonal TIV,^25,26^ male–female differences were not observed among either adult or aged individuals in either HAI seroconversion (Fig. 1b) or seroconversion rate (i.e., the proportion of individuals with post-vaccination titers that were at least 4-fold higher than pre-vaccination titers, Fig. 1c) following receipt of the monovalent 2009 H1N1 vaccine. The impact of age on HAI antibody titers, however, was gender-dependent because only among females, did adults have greater seroconversion than aged individuals (Fig. 1b, *p* *<* 0.05, one-way ANOVA).

To evaluate the functional antibody response, we analyzed 2009 H1N1 virus-specific neutralizing antibody titers by microneutralization assay, which measures the ability of serum antibodies to prevent infection of mammalian cells in vitro.^27^ Similar to HAI seroconversion, adult females had greater seroconversion as compared to aged females (Fig. 1d, *p* *<* 0.05, one-way ANOVA). The neutralizing seroconversion rate was also greater in adult females as compared to either adult males or aged females (Fig. 1e, *p* *<* 0.05, Chi square). Regardless of the methods used to assess antibody responses to the vaccine, no male–female differences were observed in seroconversion or the seroconversion rate within the population of aged individuals, and age-associated differences were only observed among females.

To analyze the predictive strength of gender and age on neutralizing antibody responses, we developed a best-fit regression model to consider the broader association of self-reported epidemiological factors that might also impact neutralizing antibody responses, including endocrine disorders (e.g., hypothyroidism), sex hormone modifications (e.g., hysterectomy [with no mention of concurrent ovariectomy], menopause, oral contraceptive use), mental health conditions (e.g., depression and anxiety), smoking, or known respiratory diseases. The regression analysis indicated that only age, gender, the interaction between age and gender, and the interaction between age and chronic respiratory disease or smoking contributed significantly to variation in the neutralizing antibody responses to the vaccine (Table 2, *p* *<* 0.05 in each case, linear regression).

**Table 2:** Predictors of neutralizing antibody titers in human males and females estimated using the best-fit model

Epidemiological factor	DF	*F* value	*p*-Value
Hypothyroidism	1, 158	0.0841	0.7722
Hysterectomy/vasectomy/post-menopausal	1, 158	0.0004	0.9849
Oral contraceptive use	1, 158	0.2542	0.6148
Depression and/or anxiety	1, 158	0.3081	0.5796
Corticosteroids	1, 158	0.1883	0.6649
Age	35, 158	2.1267	0.0009*
Gender	1, 158	4.5181	0.0351*
Chronic respiratory disease or smoker	1, 158	1.9411	0.1655
Hysterectomy/vasectomy/post-menopausal × oral contraceptive use	1, 158	0.0058	0.9397
Age × gender	16, 158	3.6545	1.15E−05*
Age × chronic respiratory disease or smoker	4, 158	3.5479	0.0084*

Model AIC value = 3033.339 (model > 10 AIC values less than other models)

*Statistically significant effect at *p* < 0.05

### Circulating sex steroids are associated with age-associated changes in neutralizing antibody responses following vaccination in humans

Reduction in the production of sex steroid hormones (i.e., estradiol and progesterone in females and testosterone in males) contributes to the age-associated dysregulation of immune function.^4^ To evaluate the associations between sex steroid hormones and influenza-specific neutralizing antibody responses in humans, we measured serum estradiol in females and serum testosterone in males 42 days post vaccination (dpv). Estradiol concentrations in serum were significantly lower in aged as compared with adult females (Fig. 1f, *p* < 0.05, *t*-test). The concentrations of estradiol in both aged and adult females were correlated with their neutralizing antibody seroconversion (Fig. 1g, *p* < 0.05, linear regression), in which females with greater estradiol concentrations had greater neutralizing antibody seroconversion, regardless of age. This correlation between estradiol concentration and neutralizing antibody response was stronger in aged (*r*^2^ = 0.80) than young (*r*^2^ = 0.38) females. Among males, aged males had significantly lower serum testosterone levels than their younger adult counterparts (Fig. 1h, *p* < 0.05, *t*-test). A negative correlation was observed between serum testosterone levels and neutralizing antibody seroconversion in adult, but not aged, males (Fig. 1i, *p* < 0.05, linear regression). Together, these data suggest that there may be a stimulatory effect of estrogen and suppressive effect of testosterone on vaccine-induced antibody responses in humans.

### Females have greater quality and quantity of vaccine-induced antibody than males among adult, but not aged, mice

**Fig. 2:**
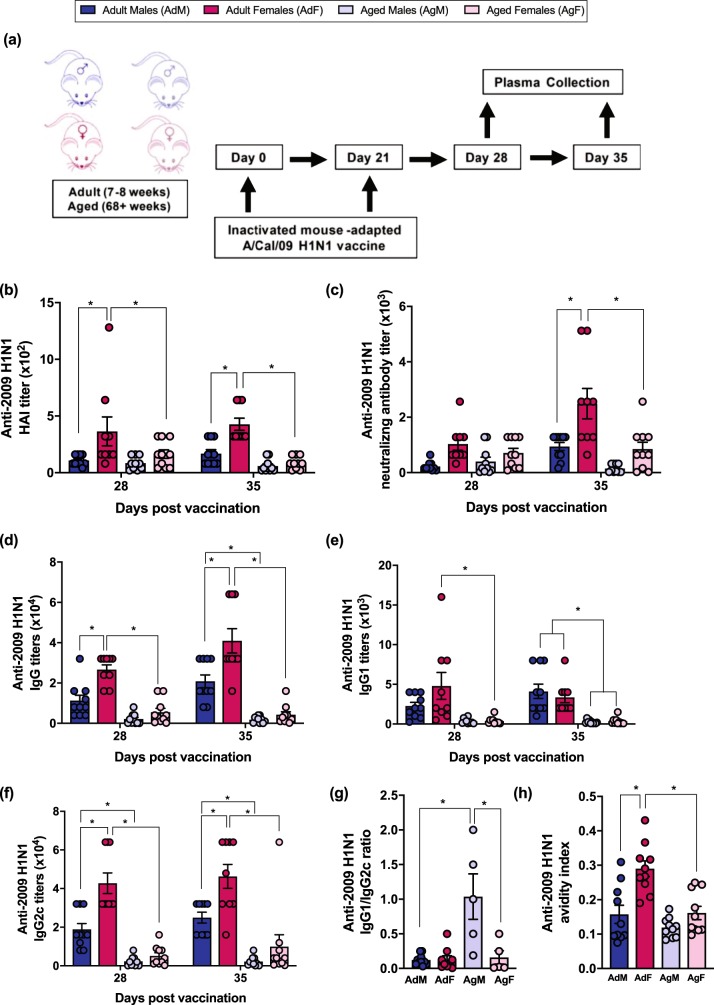
Adult female mice have greater antibody responses to an inactivated 2009 H1N1 influenza vaccine than their male counterparts, which is mitigated in aged mice. Adult (8–10 weeks) and aged (68–70 weeks) male (dark or light blue, respectively) and female (dark or light pink, respectively) mice were vaccinated with an inactivated ma2009 H1N1 vaccine and plasma was collected on days 28 and 35 (i.e., 7 and 14 days post boost) (**a**). Hemagglutination inhibition (HAI) titers (**b**), neutralizing antibody titers (**c**), anti-2009 H1N1 IgG (**d**), anti-2009 H1N1 IgG1 (**e**), anti-2009 H1N1 IgG2c (**f**), the ratio of IgG1/IgG2 (**g**), and antibody avidity (**h**) responses were measured. Data represent mean ± standard error of the mean from two independent replications (*n* = 9–10/group) and significant differences between groups are denoted by asterisks (*p < 0.05) based on two-way ANOVAs (**b**–**f**) or one-way ANOVAs (**g**, **h**)

To further characterize how age and biological sex affect the humoral immune response to influenza vaccination, adult and aged male and female mice were vaccinated with two doses of inactivated 2009 H1N1 vaccine and antibody titers were measured (Fig. 2a). Consistent with previous studies in mice,^18^ adult females developed greater 2009 H1N1-specific HAI titers than either adult males or aged females at 28 and 35 dpv (Fig. 2b, *p* < 0.05, two-way ANOVA). Adult female mice also had greater 2009 H1N1-specific neutralizing antibody titers than either adult males or aged females at 35 dpv (Fig. 2c, *p* < 0.05, two-way ANOVA). In contrast, no age-associated changes in either 2009 H1N1-specific HAI or neutralizing antibody titers were observed among male mice (Fig. 2b, c). Furthermore, there were no sex differences in vaccine-induced antibody responses among aged mice.

To begin to assess sex and age differences in the quality of vaccine-induced antibodies, anti-2009 H1N1 IgG and IgG isotypes, as well as IgG avidity, were measured. Total anti-2009 H1N1 IgG titers were greater in adult females than either adult male or aged female mice at 28 and 35 dpv (Fig. 2d, *p* < 0.05, two-way ANOVA). IgG isotypes are of particular interest because they differ in their avidity to influenza viruses. IgG2c has greater avidity than IgG1 and is associated with better influenza vaccine efficacy in mice.^28^ Overall, adult mice had greater anti-2009 H1N1 IgG1 titers than aged mice at 35 dpv (Fig. 2e, *p* < 0.05, two-way ANOVA), but sex differences in IgG1 were not observed among either adult or aged mice. In contrast, not only did adult mice have greater anti-2009 H1N1 IgG2c titers than aged mice, titers were significantly greater among adult females than either adult males or aged females at 28 and 35 dpv (Fig. 2f, *p* < 0.05, two-way ANOVA). Sex differences in IgG2c titers were not apparent among aged mice. The IgG1/IgG2c ratio, which is indicative of Th2/Th1 skewing, did not differ between the sexes in the adult mice (Fig. 2g, *p* < 0.05, one-way ANOVA). Aged male mice, however, had a higher IgG1/IgG2c ratio than either aged females or adult males (Fig. 2g, *p* < 0.05, one-way ANOVA). Adult female mice also had a greater IgG avidity index than either adult male or aged female mice (Fig. 2h, *p* < 0.05, one-way ANOVA). Taken together, these data suggest that sex differences in antibody quantity and quality following vaccination are more pronounced among adult than aged mice.

### Vaccinated females are better protected against influenza virus challenge than males among adult, but not aged, mice

**Fig. 3:**
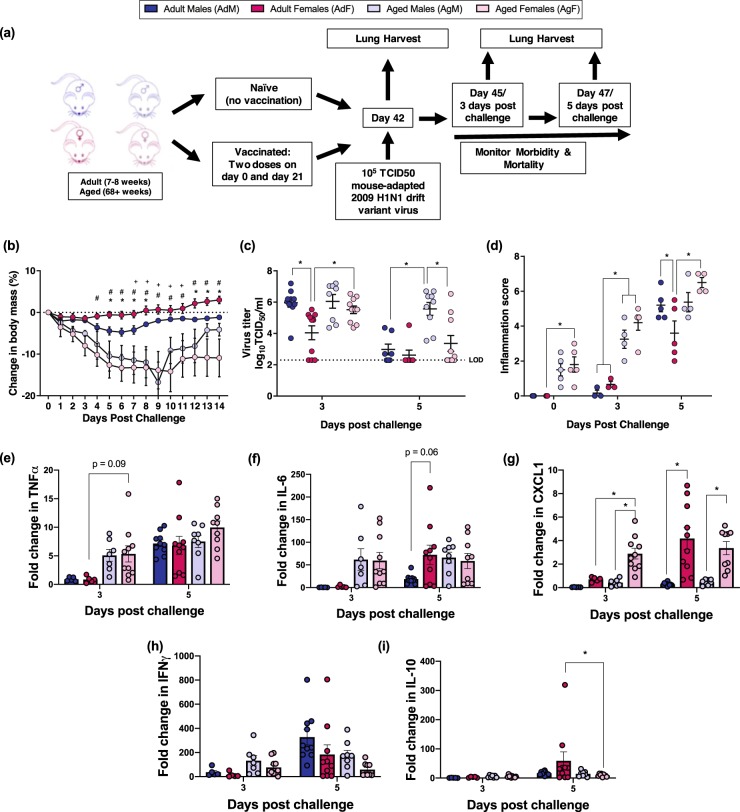
Sex differences in influenza vaccine-induced protection is more pronounced in adult than aged mice. Adult (8–10 weeks) and aged (68–70 weeks) male (dark or light blue, respectively) and female (dark or light pink, respectively) mice were vaccinated with an inactivated ma2009 H1N1 vaccine and 6 weeks post vaccination challenged with an ma2009 drift variant virus (**a**). To evaluate morbidity, the percent change in body mass was measured daily for 14 days post challenge (*n* = 9–10/group) (**b**). Lung virus titers were measured on days 3 and 5 post challenge (*n* = 9–10/group) (**c**), and cumulative inflammation scores from H&E stained lung sections were quantified prior to, and on days 3 and 5 post challenge (**d**, **e**) (*n* = 4–5/group). The concentrations of proinflammatory cytokines were assessed prior to and 3 and 5 days post challenge, and the induction (i.e., fold change) from baseline of TNFα (**e**), IL-6 (**f**), CXCL1 (**g**), IFNγ (**h**), and IL-10 (**i**) are presented. Data represents means ± standard error of the mean from two independent replications, and significant differences between groups are denoted by asterisks (*p < 0.05) based on a repeat measures ANOVA (**b**) or two-way ANOVAs (**d**–**i**). Significant differences in morbidity (**b**) between adult males and females are denoted by asterisks (*), between adult and aged females by pound sign (#), and between adult and aged males by plus sign (+); *p* < 0.05 based on a repeat measures ANOVA

To evaluate sex and age differences in influenza vaccine efficacy, adult and aged male and female mice were either unvaccinated or vaccinated with inactivated 2009 H1N1 and challenged 6 weeks later with a drift variant 2009 H1N1 virus (2009 H1N1dv) (Fig. 3a). Among vaccinated mice, adult mice were better protected against challenge with the 2009 H1N1dv virus, experiencing less morbidity and clearing influenza virus from their lungs faster than aged mice (Fig. 3b, c, *p* < 0.05 in each case, repeat measures ANOVA or two-way ANOVA, respectively). Among adult mice, vaccinated females were better protected following 2009 H1N1dv virus challenge, losing significantly less body mass than vaccinated males (Fig. 3b, *p* < 0.05, repeat measures ANOVA). Vaccinated adult females also cleared virus from their lungs faster than either vaccinated adult males or aged females (Fig. 3c, *p* < 0.05, two-way ANOVA). Sex differences in protection against challenge were not observed among aged mice. These data suggest that sex-specific differences in vaccine efficacy are present among adult, but not aged, mice.

Among young adult mice, regardless of sex, vaccinated mice were better protected against challenge with the 2009 H1N1dv virus experiencing less body mass loss and having lower pulmonary virus titers than unvaccinated adult mice (Supplementary Fig. 1a and 1c, *p* < 0.05 in each case, two-way ANOVAs). In contrast, among aged mice, vaccinated males and females suffered body mass loss that was comparable to unvaccinated mice (Supplementary Fig. 1b). Vaccinated aged females, however, had lower virus titers after challenge than unvaccinated aged females, suggesting protection following vaccination in aged female, but not male, mice (Supplementary Fig. 1d, *p* < 0.05, two-way ANOVA). These data further suggest that vaccine-induced protection was greater among adult than aged mice and greater in female as compared with male mice.

Because vaccinated aged mice, both males and females, suffered significantly greater morbidity than adult vaccinated mice following virus challenge, we sought to assess vaccine-induced protection from pulmonary inflammation caused by the challenge virus. Lung tissue excised from vaccinated mice was analyzed for markers of inflammation prior to challenge and 3 or 5 days post 2009 H1N1dv virus challenge. Vaccinated aged mice had evidence of pulmonary inflammation, including perivascular inflammation and peribronchiolar inflammation, prior to 2009 H1N1dv virus challenge, which was not apparent among adult mice (Fig. 3d, *p* < 0.05, two-way ANOVA). After virus challenge, pulmonary inflammation, including alveolar inflammation, peribronchiolar inflammation, perivascular inflammation, and edema significantly increased 3 and 5 days post challenge in aged male and female mice (Fig. 3d, *p* < 0.05, two-way ANOVA). Pulmonary inflammation remained low among vaccinated adult male and female mice, with an increase 5 days post challenge. Inflammation at 5 days post challenge, however, was significantly lower for adult females than for adult male or aged female mice (Fig. 3d, *p* < 0.05, two-way ANOVA). These data suggest that vaccine-induced protection from pulmonary inflammation and tissue damage after challenge was greatest for adult females.

We also evaluated pulmonary concentrations of inflammatory proteins (i.e., IFN-γ, IL-6, CXCL1, IL-10, and TNFα) in vaccinated and unvaccinated mice 0, 3, and 5 days post challenge. Among vaccinated mice, there were few discernable patterns in either the concentrations (Supplementary Tables 1 and 2) or induction of cytokines and chemokines in the lungs after virus challenge (Fig. 3e–i). Among vaccinated young adults, females had a greater induction of IL-6 and CXCL1 than their male counterparts (Fig. 3f, g, *p* = 0.06 and *p* < 0.05, respectively, two-way ANOVA). Overall, vaccinated aged mice exhibited an earlier induction of the pulmonary cytokines than vaccinated adult mice (Fig. 3e–g).

To confirm that vaccination reduced the induction of pulmonary inflammatory proteins, especially among aged mice, we compared cytokine and chemokine responses among vaccinated and unvaccinated adult and aged male and female mice. Overall, pro-inflammatory cytokines associated with the acute phase response, including TNFα and IL-6, showed greater induction in unvaccinated than vaccinated mice, regardless of age (Supplementary Fig. 2a–2d). In unvaccinated aged mice, males had greater induction of TNFα and IL-6 than females (Supplementary Fig. 2b and 2d, *p* < 0.05 in each case, two-way ANOVAs). Vaccinated mice had greater induction of IFNγ and IL-10 than unvaccinated mice, with young mice showing greater induction of these cytokines (Supplementary Fig. 2g–2j). Taken together, these data suggest that vaccination reduced the acute phase response but increased the induction of T cell-associated cytokines, regardless of the age or sex of the mice. Because this pattern of cytokine and chemokine activation did not match the pattern of protection against virus challenge, we interpret these data to suggest that cytokines do not mediate how inactivated vaccine-induced immunity differentially protects young adult female as compared with either young adult male or aged female mice.

### Sex steroid hormones modulate vaccine-specific antibody responses differently for male and female mice

**Fig. 4:**
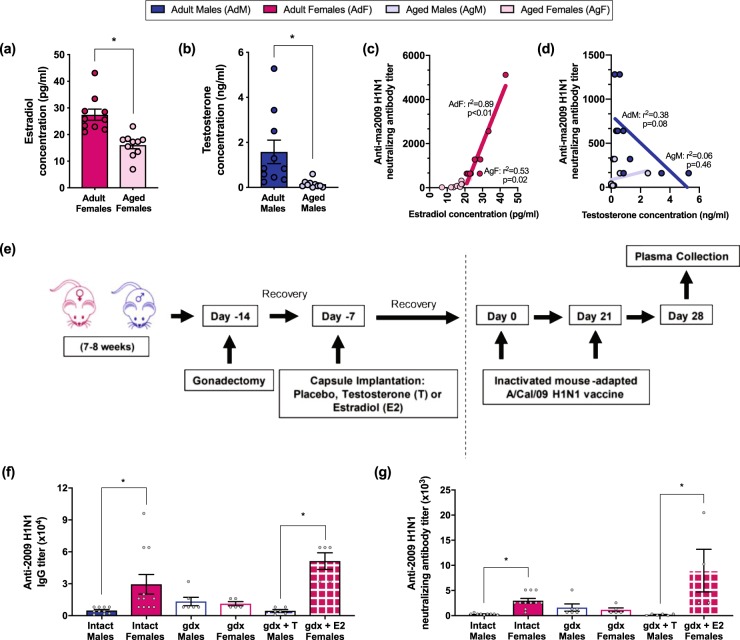
Estradiol in female mice increases, whereas testosterone in male mice decreases, antibody titers following vaccination. Adult (8–10 weeks) and aged (68–70 weeks) male (closed, opened, or stippled blue) and female (closed, open, or stippled pink) mice were vaccinated with an inactivated ma2009 H1N1 vaccine and 6 weeks post vaccination plasma estradiol and testosterone levels were measured (**a**, **b**) and correlated with neutralizing antibody responses quantified by a linear regression model (**c**, **d**) (*n* = 10/group). Female and male mice were gonadectomized (gdx), implanted with either estradiol (females, gdx + E2), testosterone (males, gdx + T) or placebo capsules, and provided 1 week to recover from surgery prior to vaccination with an inactivated ma2009 H1N1 vaccine (**e**). Plasma anti-2009 H1N1 IgG (**f**) and neutralizing antibody (**g**) titers were measured in gonad intact, gdx, gdx + T, and gdx + E2 mice at 35 days post vaccination (*n* = 5/treatment group). Data represent means ± standard error of the mean from two independent replications and significant differences between groups are denoted by asterisks (*p < 0.05) based on *t*-tests (**a**, **b**), regression analyses (**c**, **d**), or one-way ANOVAs (**f**, **g**)

To evaluate whether circulating sex steroid concentrations were affected by age in a similar manner in mice as in humans (Fig. 1f, h), concentrations of estradiol in females and testosterone in males were measured at 42 dpv. As in the human subjects, aged female mice had lower plasma estradiol than younger adult females and aged male mice had lower plasma testosterone than younger adult males (Fig. 4a, b, *p* < 0.05 in each case, *t*-tests). A strong positive correlation between plasma estradiol levels and neutralizing antibody titers was observed among females (Fig. 4c, *p* < 0.05, *r*^2^ = 0.89 in adult females, *r*^2^ = 0.53 in aged females, linear regression). Among males, lower plasma testosterone tended to be associated with increased neutralizing antibody titers in adult but not aged mice (Fig. 4d, *p* = 0.08, *r*^2^ = 0.38, linear regression).

To determine whether estrogens in females and androgens in males cause sex differential antibody responses following vaccination, we removed the gonads in adult mice, and in subsets of animals replaced estradiol in females and testosterone in males (Fig. 4e). Removal of the gonads eliminated sex differences in both anti-2009 H1N1 IgG and neutralizing antibody titers at 35 dpv (Fig. 4f, g). Among females, gonadectomized (gdx) mice tended to have lower IgG and neutralizing antibody titers than intact females, whereas gdx females that received exogenous estradiol had significantly greater IgG and neutralizing antibody titers as compared to gdx females that received placebo (Fig. 4f, g, *p* < 0.05 in each case, one-way ANOVAs). Among males, gdx males had significantly greater anti-2009 H1N1 IgG and neutralizing antibody titers than either intact males or gdx males that received exogenous testosterone (Fig. 4f, g, *p* < 0.05 in each case, one-way ANOVAs). Replacement of testosterone in gdx males significantly reduced anti-2009 H1N1 IgG and neutralizing antibody titers as compared to gdx males treated with placebo (Fig. 4f, g, *p* < 0.05 in each case, one-way ANOVAs). Taken together, these data illustrate that among adult mice, estradiol enhances vaccine-induced antibody responses in females, whereas testosterone reduces vaccine-induced antibody responses in males.

## Discussion

Immune responses to vaccines decrease with age, but whether this occurs similarly in males and females or whether it is associated with reproductive senescence has not been addressed. Utilizing serum samples from an NIH-sponsored trial of a monovalent 2009 H1N1 vaccine,^29^ we evaluated influenza vaccine-induced sex/gender differences in adults of reproductive (i.e., 18–45 years) and non-reproductive (i.e., 65+ years) ages. We further delineated the impact of sex, age, and reproductive hormones on influenza vaccine-induced responses and protection using a mouse model that allowed for greater manipulation to determine causality of host responses. The major findings from these studies were (1) in humans, self-reported gender and age were significant predictors of variation in neutralizing antibody responses against the 2009 H1N1 vaccine; (2) in both vaccinated humans and mice, adult females had greater neutralizing antibody responses than males, which was diminished in aged individuals; (3) the impact of aging on reducing antibody responses was greater for females than males; (4) the steroid hormonal milieu affected vaccine-induced antibody responses in males and females of both humans and mice; and (5) female-biased antibody responses to the influenza vaccine resulted in greater protection from influenza virus infection in mice.

Sex/gender differences in response to both pandemic and seasonal influenza vaccines in humans have been documented, in which females typically generate greater vaccine-induced antibody responses than males.^30^ Whether female-biased immunity following influenza vaccination is maintained across the life course is rarely considered. Further, the assays used to evaluate influenza vaccine-induced antibody responses may yield differential findings, which also is seldom addressed. In the current study, sex/gender differences in HAI and neutralizing antibody titers were more prominent in younger than older adults among both humans and mice. In humans, with pre-existing immunity, the measurement of HAI seroconversion rates dampened both self-reported gender and age-associated differences that were apparent for neutralizing antibody seroconversion rates. These findings raise questions about the ability of HAI to accurately measure variation in antibody responses among biologically distinct groups of individuals and suggest that microneutralization assays may be more sensitive.

Consistent with humans, female mice generate greater antibody responses to influenza vaccines as compared to male mice.^18,20,21^ To date, all mouse studies have utilized young adult animals of reproductive ages to assess sex differences in vaccine-induced immunity. In the current study, sex differences in vaccine-induced immunity and protection were mitigated in aged mice. Vaccine-induced IgG, IgG2c, and neutralizing antibody responses were consistently greater in adult females as compared to adult males. In contrast, sex differences in vaccine-induced antibody responses were not observed among aged mice, which is primarily explained by the greater influence of aging on immune responses in females as compared with males. In addition to the quantity of vaccine-induced antibody, we found that the quality of the antibodies also differed by sex and age in mice. Anti-influenza IgG2c antibodies are associated with a Th1 response, play an important role in viral clearance,^28^ and are greater in adult females than males. Adult female mice also developed antibodies against influenza virus that were of greater avidity than those of the males. These data are in contrast with human studies where adult females developed lower avidity influenza-specific antibodies compared to adult males, suggesting that antibody avidity may be species-specific or dependent on differences in preexisting immunity.^31^ Although a skewing of Th1/Th2 responses is associated with aging,^32^ studies investigating these responses have not considered the sex of the animals.^33^ In the current study, the anti-influenza IgG1/IgG2c (Th2/Th1) ratio did not differ between adult males and females; among aged mice, however, males had a greater IgG1/IgG2c ratio than females.

Analysis of cytokine responses in humans has been used to predict the immunogenicity of vaccines.^34^ In our study, serum IL-6 concentrations increased with vaccination in young adult females only. IL-6 promotes T-follicular helper cell and activated B cell differentiation.^35^ Although sex differences in serum IL-6 have not been investigated in the context of influenza vaccinations, previous work has shown that females have a greater rise in IL-6 from baseline compared to males following stress tasks.^36^ These data may suggest a potential role for IL-6 in sex-specific immune responses.

The observation that sex differences in vaccine-induced immune responses and protection were reduced in aged individuals suggests that age-related changes in sex steroid hormones may be involved. Among both humans and mice, aged females and males had lower circulating concentrations of estradiol and testosterone, respectively, as compared with their younger adult counterparts. The observation that serum estradiol in females of all ages correlated with seroconversion to a monovalent 2009 H1N1 vaccine, whereas serum testosterone levels negatively correlated with seroconversion in adult, but not aged, males requires further consideration. Aging is associated with dramatic changes in the sex steroid milieu in both males and females, where serum testosterone levels in males undergo a slow, physiological decline, and serum estradiol levels in females decline more rapidly.^4^ The association between serum estradiol and antibody responses following seasonal influenza vaccination has been previously evaluated, in which post-menopausal females that received hormone therapy developed antibody responses that were similar to those of females not on hormone therapy.^37,38^ Conversely, other studies have illustrated that the use of hormone replacement therapy in post-menopausal females is associated with increased numbers of circulating B cells and decreased levels of proinflammatory cytokines.^39,40^ Using a systems biology approach to evaluate the contribution of serum testosterone to TIV-induced antibody responses, males with the highest serum testosterone levels had the lowest influenza-specific antibody responses,^14^ but age-associated changes in testosterone were not considered.

In the current study, variability in the concentration of sex steroid hormones was associated with differences in vaccine-induced antibody responses in both humans and mice. In humans, however, sex steroid modifications associated with hysterectomy (without complete documentation of concurrent ovariectomy), menopause, or oral contraceptive use were not independently predictive of variability in vaccine-induced immunity. Among females, serum estradiol concentrations were positively correlated with seroconversion in both adult and aged individuals and partially explain reduced vaccine-induced immunity and protection from influenza with aging in females. Among males, circulating testosterone concentrations negatively correlated with seroconversion in adults of reproductive ages only. The association between testosterone concentrations and antibody responses changed with age in males, in which higher testosterone concentrations were associated with higher antibody responses among aged humans and mice. Few studies have considered the association between sex steroid hormones and immunity to vaccines.

A previous report from our laboratory demonstrated no effect of testosterone on influenza-specific antibody responses following infection with live virus, which may reflect a fundamental difference in the impact of testosterone on immunity to influenza induced by infection versus vaccination.^41^ Previous studies illustrate that removal of the ovaries reduces whereas exogenous treatment with estradiol increases antibody responses following influenza infection.^42^ These data are consistent with our observation that higher serum estradiol concentrations cause greater vaccine-induced antibody responses. The mechanisms mediating how sex steroids affect antibody responses remain to be determined. Sex steroid hormone signaling may alter the production of antibodies by B cells, including through altered help from T cell subsets (e.g., Th1 cells).

There are limitations, however, to the current study ﻿that should be considered when interpreting our findings. Unlike previous studies investigating the effects of sex/gender and age on immune responses in humans, here we partitioned the population by reproductive status. Although this was an important aspect of our work, doing so resulted in small sample sizes and lower power to identify small changes in immune responses. Future research would benefit from designing an a priori study to more rigorously analyze the influence of sex and age on vaccine-induced immune responses in a larger population. Despite these limitations, regression models revealed that age and gender more so than other variables impact vaccine-induced antibody responses.

In summary, our studies in both humans and mice provide evidence that the impact of sex/gender is age-dependent for vaccine-induced antibody responses and protection against influenza. We show that antibody responses are associated with concentrations of reproductive hormones in both sexes, but the impact of aging on immunity to influenza is greater for females than males. These data suggest that experimental design and data analyses should consider sex/gender and age in preclinical animal studies, as well as in randomized controlled trials of influenza vaccines. Together, the results of this study contribute to our understanding of how sex/gender and age influence the efficacy of influenza vaccines and highlight important areas of further research to investigate the mechanisms by which the immune response differs across populations.

## Methods

### Human serology

Human serum samples were obtained through a clinical trial (NCT00943488) from the National Institutes of Health Division of Microbiology and Infectious Diseases (NIH-DMID) Center for Excellence in Influenza Research & Surveillance (CEIRS) network.^29^ Adult males and females were vaccinated with two doses (30 μg each, 21 days apart) of inactivated monovalent unadjuvanted split-virus H1N1 A/California/07/09 NYMC-X-179A vaccine (CSL Biotherapies). Serum was collected prior to vaccination and 21 days post boost (days 0 and 42). For our analysis, males and females were stratified into two age groups (18–45 years: *n* = 20 adult males, 30 adult females, and 65+ years: *n* = 47 aged males, and 48 aged females) with sample sizes based on the availability of samples within the requested age ranges. Because of the nature of the study, individuals could not be randomly assigned based on sex or age, but all samples were numbered and investigators were blinded to groups during serum sample processing. Seroconversion was calculated by dividing the post and pre-vaccination antibody titers and seroconversion rate was defined as the rate of individuals with at least a 4-fold increase in antibody titers.

### Animals

All animal procedures were approved by the Johns Hopkins University Animal Care and Use Committee (MO15H236). Adult (8–10 weeks) and aged (68–70 weeks) male and female C57BL/6J mice were purchased from Jackson Laboratories and housed at 3–5 animals per cage. The mice were housed under standard biosafety level 2 housing with food and water provided ad libitum. Mice were acclimated to the facility for at least 1 week prior to starting experiments. For studies of sex and age differences, animals could not be randomly assigned to groups, but for hormone treatment studies, animals were randomly assigned to treatment groups. All samples were numbered and investigators were blinded to groups during sample processing.

### Vaccination and challenge

Mouse-adapted A/California/04/09 (ma2009 H1N1) was generated by reverse genetics from a published sequence.^43^ For vaccination, ma2009 was inactivated by Beta propiolactone and confirmed by TCID_50_ assay. Mice were vaccinated intramuscularly with 20 μg of inactivated ma2009 H1N1 or vehicle alone on days 0 and 21 using a prime-boost strategy. Mice were challenged on day 42 post vaccination with 10^5^ TCID_50_ units of a mouse-adapted A/California/04/09 drift variant virus (ma2009dv) containing a K166Q mutation in the HA sequence (kindly provided by Dr. Andrew Pekosz, Johns Hopkins University).^44^ Mice were then monitored for 2 weeks or euthanized at 3 and 5 days post challenge to collect blood and lung tissue.

### Gonadectomy and hormone replacement

Male and female mice (8–10 weeks) were either bilaterally gonadectomized (gdx) or left intact with a sham surgery.^42,45^ After a 2-week recovery period, mice were implanted subcutaneously with a silastic capsule (inner diameter—0.04″, outer diameter—0.085″) filled with either testosterone (T; 7.5 mm) for males or 17β-estradiol for females (E2; 5 mm) or left empty (placebo).^41^ The capsules were sealed with 2.5 mm of medical adhesive (Factor II, A-100), incubated at 37 °C overnight in sterile saline solution prior to implantation and were replaced every 21 days.

### Hemagglutinin inhibition assay

HAI antibody titers from the human serum samples were provided and published previously,^29^ but had not previously been analyzed for sex and age-associated differences. Mouse plasma samples were heat inactivated at 56 °C for 30 min, diluted with 100 μl of 5% sodium citrate and sterile normal sodium chloride solution to obtain a final dilution of 1:20, incubated at 37 °C for 30 min and cooled to room temperature. One drop (~50 μl) of packed turkey red blood cells (RBC; Innovative Research) was added to the sample, centrifuged at 2000 rpm for 10 min and the supernatant was collected. Hemagglutination assay (HA) was performed by serially diluting 25 μl of virus (ma2009 H1N1) across a round-bottom 96-well plate in 25 μl of 0.01 M PBS. 50 μl of a 0.5% RBC suspension was added to the virus dilutions and incubated at room temperature for 2 h. The HA titer was determined by the highest dilution causing complete agglutination. For the hemagglutination inhibition assay (HAI), mouse plasma was serially diluted across the plate, 25 μl of 4× HA virus was added to each well and incubated at room temperature for 1 h. 50 μl of a 0.5% RBC suspension was added to each well and allowed to agglutinate at room temperature for 2 h. The HAI titer was calculated as the reciprocal of the highest dilution causing a complete inhibition of agglutination.

### Microneutralization assay

Human serum samples were mixed with receptor destroying enzyme (RDE, Denka Seiken Co. Ltd.) at a 1:3 ratio, incubated overnight at 37 °C, and then heat inactivated at 57 °C for 30 min. Mouse plasmas was heat inactivated at 57 °C for 30 min. Human serum or mouse plasma was serially diluted in infection media (Dulbecco’s Modified Eagle’s Medium (DMEM) in the presence of penicillin, streptomycin, 0.5% BSA, and N-acetyl Trypsin 5 μg/ml), mixed with 100 TCID_50_ of virus (A/California/07/2009 for humans or ma2009 H1N1 for mice) and incubated for 1 h at room temperature. The serum/plasma and virus mixture was then used to infect quadruplicate wells of confluent Madin-Darby Canine Kidney (MDCK) cells for 24 h at 37 °C (human) or 32 °C (mouse). Following the 24-h incubation, the inoculum was removed, and the cells were washed one time with 1× PBS and new infection media was added. The cells were incubated at 32 °C until complete cytopathic effect was observed, fixed with the addition of 4% formaldehyde, and stained with naphthol blue black at room temperature overnight. The neutralizing antibody titer was calculated as the highest serum/plasma dilution that eliminated virus cytopathic effects in 50% of the wells.

### Anti-influenza virus enzyme-linked immunosorbent assays (ELISA)

ELISA plates (Greiner Bio-One) were coated with 100 ng of purified ma2009 H1N1 virus protein in carbonate–bicarbonate buffer (pH 9.6) and incubated at 4 °C overnight. Plates were washed three times with wash buffer (1× PBS + 0.1% Tween-20), blocked with 10% milk in 1× PBS solution and incubated for 1 h at 37 °C. Serially diluted serum/plasma samples were added, and the plates were incubated at 37 °C for 1 h. The plates were washed three times, secondary antibody (IgG—1:250 [ThermoFisher 32430], IgG1—1:8000 [ThermoFisher PA1-74421], or IgG2c—1:30,000 [ThermoFisher PA1-29288]) was added, and the plates were incubated for 1 h at 37 °C. The plates were washed three times and reactions were developed with 3,3′,5,5′- tetramethylbenzidine (TMB, Fisher Scientific) for 20 min. The reaction was stopped using 1 N HCl. Plates were read at 405 nm absorbance and antibody titers were calculated as the highest serum/plasma dilution with an OD value above 3 times the average OD of the negative controls.^44^

### Anti-influenza virus avidity

ELISA plates (Greiner Bio-One) were coated with 100 ng of purified ma2009 virus protein in carbonate–bicarbonate buffer (pH 9.6) and incubated at 4 °C overnight. After washing with wash buffer, the plates were blocked with 10% milk in 1× PBS solution and incubated for 1 h at 37 °C. Plasma samples were plated in quadruplicate at a 1:60 dilution in sample buffer (5% FBS in 1× PBS) and incubated for 1 h at 37 °C. To measure antibody avidity, ammonium thiocyanate (NH_4_SCN; 2 M—mouse, 3M—human; Sigma) or 1× PBS was added to the plates for exactly 15 min. The plates were washed eight times with wash buffer and IgG secondary antibody (ThermoFisher Scientific) was added and incubated for 1 h at 37 °C. The plates were washed three times with wash buffer and reactions were developed with TMB for 20 min. The reaction was stopped using 1 N HCl. Plates were read at 450 nm absorbance and the antibody avidity index was determined by normalizing the NH_4_SCN treated absorbance values to the corresponding 1× PBS (untreated) values for each sample in duplicate.

### Steroid hormone enzyme-linked immunoassays (ELISAs)

Circulating estradiol and testosterone concentrations in human serum and mouse plasma collected at day 42 post vaccination were determined using commercial ELISA kits according to manufacturer’s instructions (Calbiotech—estradiol; IBL America—testosterone).

### TCID_50_ virus quantification assay

Frozen lung samples were homogenized in DMEM, centrifuged at 2500 rpm for 10 min at 4 °C and the supernatant was collected. Ten-fold serial dilutions of lung homogenates were made in infection media and were plated onto confluent MDCK cells in six replicates. The plates were incubated for 6 days at 32 °C. The cells were then fixed with 4% formaldehyde and stained with naphthol blue black overnight. The cytopathic effect was scored visually and 50% tissue culture infective dose was determined by the Reed–Muench method.^46^

### Multiplex cytokine assays

Inflammatory and immune response cytokines (IFN-γ, IL-1β, IL-2, IL-4, IL-6, IL-8, IL-10, IL-12p70, IL-13, and TNF-α) were measured in human serum samples in singlicate using the V-PLEX Proinflammatory Panel 1 Human Kit (Meso Scale Discovery, Gaithersburg, MD) according to manufacturer protocol. From mouse lung supernatants, the concentrations of cytokines (CXCL1, IFN-γ, IL-6, IL-10, and TNF-α) were measured in singlicate using the V-PLEX Proinflammatory Panel 1 Kit (Meso Scale Discovery) according to manufacturer protocol. Overall, there was low detection of cytokines prior to infection, therefore we analyzed induction.

### Histopathology

Mouse lungs were inflated under constant pressure and fixed in Z-fix (Anatech). Tissues were sectioned (5 μm), mounted on glass slides, and stained with hematoxylin and eosin (H&E) to assess lung inflammation. Blinded scoring was performed by single observer under the guidance of a veterinary pathologist on a scale of 0–3 (0—none, 1—mild, 2—moderate, and 3—severe inflammation) for the following parameters: perivascular inflammation, peribronchiolar inflammation, alveolar inflammation, and edema.^44^

### Statistics

Antibody responses, hormone concentrations, viral titers, cytokine concentrations, and inflammation scores were analyzed using *t*-tests and two-way or one-way ANOVAs followed by post-hoc multiple comparisons using Dunn–Bonferroni multiple comparison test. Proportion data (e.g., percent seroconversion rate) were analyzed using Chi-square analyses. Correlational analyses between antibody titer and steroid concentrations were analyzed using linear regression. Morbidity data were analyzed with repeated measures ANOVA and post-hoc tests were performed correcting for multiple comparisons using the Dunn–Bonferroni method.

To assess the influence of self-reported epidemiological factors (i.e., gender; ethnicity; race; autoimmune disease; hypothyroidism; chronic respiratory disease or smoking; total hysterectomy, vasectomy, post-menopausal, or polycystic ovarian syndrome; anxiety and/or depression; reproductive therapy or oral contraceptives; corticosteroid treatment; and age) on immune responses, we used ﻿generalized linear mixed effects models with subject and time point (i.e., pre or post vaccination) as fixed effects. We were not able to consider ethnicity, race, or autoimmune disease as covariates, as these factors were available in very small numbers in our sample population.^47^ We selected the model that best fit the data using model comparison with Akaike’s Information Criterion.^48^ To select the model of best-fit, we used an iterative process in which we explored a number of models, by systematically adding or deleting all explanatory variables and their interaction effects to determine a balance between parsimony of explanation and a good-fitting model.^49^ Correlations were assessed using the Pearson *r* test. Mean differences were considered statistically significant if *p* < 0.05. Statistics were performed using GraphPad Prism 7 and ﻿ R v. 3.3.3 (R Core Team, 2017).

### Reporting summary

Further information on experimental design is available in the Nature Research Reporting Summary linked to this article.

## Supplementary Information

Supplementary File

Reporting Summary

## Data Availability

There are not restrictions on data availability.

## Supplementary Information

**Supplementary information** accompanies the paper on the *npj Vaccines* website (10.1038/s41541-019-0124-6).
