# Author Correction: Age-associated changes in the impact of sex steroids on influenza vaccine responses in males and females

**DOI:** 10.1038/s41541-019-0130-8

**Published:** 2019-08-12

**Authors:** Tanvi Potluri, Ashley L. Fink, Kristyn E. Sylvia, Santosh Dhakal, Meghan S. Vermillion, Landon vom Steeg, Sharvari Deshpande, Harish Narasimhan, Sabra L. Klein

**Affiliations:** 1 W. Harry Feinstone Department of Molecular Microbiology and Immunology, Johns Hopkins University Bloomberg School of Public Health, Baltimore, MD 21205 USA; 2 Department of Molecular and Comparative Pathobiology, The Johns Hopkins School of Medicine, Baltimore, MD 21201 USA; 3 Department of Biochemistry and Molecular Biology, Johns Hopkins University Bloomberg School of Public Health, Baltimore, MD 21205 USA; 4 Present Address: Biology Department, College of Saint Benedict and Saint John’s University, Collegeville, MN 56321 USA

**Keywords:** Humoral immunity, Influenza virus

**Correction to:** *npj Vaccines* 10.1038/s41541-019-0124-6 published online 12 July 2019

In the original version of this Article, an incorrect contract number was quoted for the Johns Hopkins Center for Influenza Research and Surveillance, it should have been HHSN272201400007C. This has now been corrected in the PDF and HTML versions of the Article.

