# Supplementary material for: Age-associated changes in the impact of sex steroids on influenza vaccine responses in males and females
Source: NPJ Vaccines. 2019 Jul 12;4:29. doi: 10.1038/s41541-019-0124-6 (PMC6626024; doi:10.1038/s41541-019-0124-6)
Supplement: Supplementary file 1 — Supplementary File [file 41541_2019_124_MOESM1_ESM.docx]

**Supplementary Table 1.** Concentrations (pg/ml) of cytokines and chemokines in the lungs of adult male or female mice that were either unvaccinated or vaccinated prior to challenge with ma2009 drift variant virus. Lungs were collected either prior to (0) or 3 or 5 days post-infection (DPI), with data presented as the mean + the standard error of the mean. Different superscript letters represent statistically significant differences from the other DPI within a treatment group based on two way ANOVAs (*p*<0.05).

| **DPI** | **Groups** | **IFNγ** | **IL-10** | **IL-6** | **CXCL1** | **TNFα** |
| --- | --- | --- | --- | --- | --- | --- |
| **0** | Unvaccinated male | 0.1±0.0 | 0.4±0.1 | 2.2±0.4 | 20.7±5.9^a^ | 0.8±0.1 |
|  | Unvaccinated female | 0.1±0.0 | 0.5±0.1 | 2.3±0.3 | 14.0±0.2^a^ | 1.0±0.1 |
|  | Vaccinated male | 0.1±0.0 | 1.3±0.2 | 26.4±8.7 | 305.0±132.0^b^ | 2.5±0.4 |
|  | Vaccinated female | 0.1±0.0 | 0.4±0.2 | 2.6±0.4 | 10.6±2.2^a^ | 1.9±0.3 |
| **3** | Unvaccinated male | 1.0±0.2 | 1.3±0.2 | 756.1±106.6^a^ | 408.3±43.9^a^ | 22.5±1.5^a^ |
|  | Unvaccinated female | 1.0±0.2 | 1.6±0.1 | 726.3±148.5^a^ | 402.1±68.5^a^ | 30.4±2.3^a^ |
|  | Vaccinated male | 2.9±1.2 | 1.0±0.3 | 10.2±2.7^b^ | 11.0±1.6^b^ | 2.2±0.4^b^ |
|  | Vaccinated female | 1.8±1.1 | 1.1±0.3 | 5.1±3.0^b^ | 7.4±0.9^b^ | 1.6±0.5^b^ |
| **5** | Unvaccinated male | 1.2±0.3^a^ | 1.8±0.2^a^ | 410.7±70.1^a, b^ | 328.0±50.3^a^ | 25.4±4.2^a^ |
|  | Unvaccinated female | 1.5±0.3^a^ | 2.0±0.3^a^ | 517.6±40.3^a^ | 237.3±21.1^a^ | 37.2±7.3^b^ |
|  | Vaccinated male | 25.5±5.1^b^ | 21.9±2.9^b^ | 494.2±85.6^a^ | 85.6±12.4^b^ | 17.6±1.5^a, c^ |
|  | Vaccinated female | 22.6±9.8^b^ | 22.9±11.8^b^ | 184.8±54.7^b^ | 44.4±9.9^b^ | 12.9±3.0^c^ |

**Supplementary Table 2.** Concentrations (pg/ml) of cytokines and chemokines in the lungs of aged male or female mice that were either unvaccinated or vaccinated prior to challenge with ma2009 drift variant virus. Lungs were collected either prior to (0) or 3 or 5 days post-infection (DPI), with data presented as the mean + the standard error of the mean. Different superscript letters represent statistically significant differences from the other DPI within a treatment group based on two way ANOVAs (*p*<0.05).

| **DPI** | **Groups** | **IFNγ** | **IL-10** | **IL-6** | **CXCL1** | **TNFα** |
| --- | --- | --- | --- | --- | --- | --- |
| **0** | Unvaccinated male | 0.1±0.0 | 0.7±0.0 | 3.6±0.2 | 35.0±12.7^a^ | 1.6±0.3 |
|  | Unvaccinated female | 0.1±0.0 | 0.9±0.3 | 4.9±0.9 | 23.1±5.9^a^ | 1.8±0.1 |
|  | Vaccinated male | 0.1±0.0 | 1.1±0.1 | 5.1±0.6 | 220.8±67.8^b^ | 3.0±0.4 |
|  | Vaccinated female | 0.1±0.00 | 1.2±0.4 | 3.4±0.3 | 17.4±4.4^a^ | 2.2±0.3 |
| **3** | Unvaccinated male | 2.0±0.9^a^ | 1.7±0.3 | 505.7±90.5^a, b^ | 180.1±35.8^a^ | 20.9±3.2 |
|  | Unvaccinated female | 0.6±0.1^a^ | 2.4±0.4 | 804.6±358.2^a^ | 184.6±68.3^a^ | 14.2±4.3 |
|  | Vaccinated male | 11.4±3.7^b^ | 5.5±1.7 | 317.3±122.6^a, b^ | 92.2±24.6^a, c^ | 15.2±3.1 |
|  | Vaccinated female | 9.5±2.7^b^ | 6.7±1.5 | 200±60.2^b^ | 50.3±7.9^b, c^ | 11.6±3.1 |
| **5** | Unvaccinated male | 1.6±0.5^a^ | 2.8±0.5^a^ | 1375.1±217.9^a^ | 364.5±42.8^a^ | 45.6±5.3^a^ |
|  | Unvaccinated female | 1.6±0.7^a^ | 4.0±0.8^a^ | 671.4±134.2^b^ | 218.9±56.0^b^ | 28.7±4.4^b^ |
|  | Vaccinated male | 15.3±3.6^b^ | 15.7±3.6^b^ | 339.9±62.5^b^ | 102.9±17.7^b, c^ | 22.4±3.2^b^ |
|  | Vaccinated female | 7.2±2.3^a^ | 11.1±1.9^b^ | 196.8±56.1^b^ | 58.5±9.8^c^ | 21.7±2.5^b^ |

**
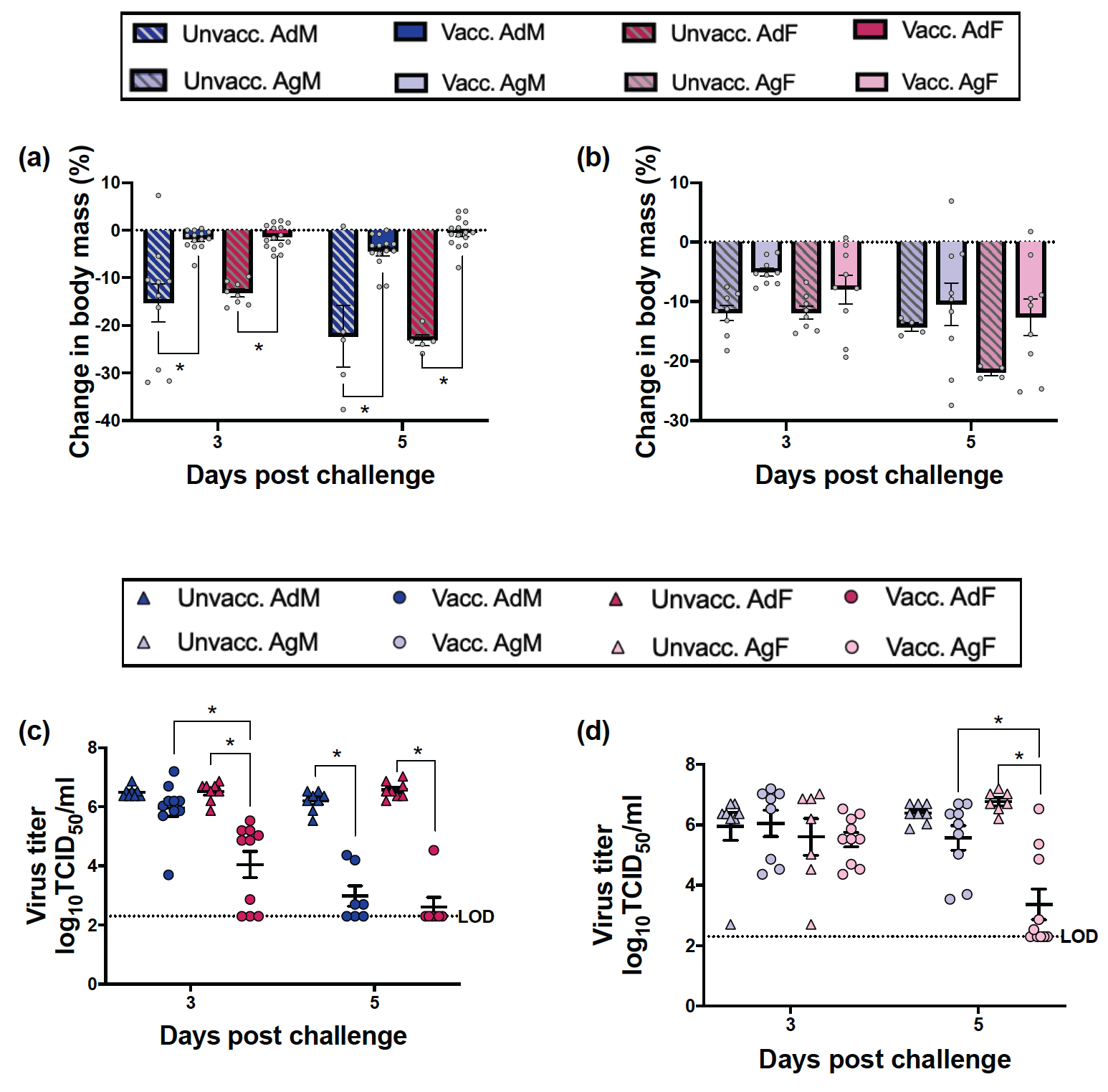
Supplementary Figure 1: Vaccinated mice, regardless of sex or age, were better protected against challenge with the 2009 H1N1dv virus.**

Vaccinated (Vacc) and unvaccinated (Unvacc) adult (8-10 weeks) and aged (68-70 weeks) male (dark [adult] and light [aged] blue, solid [Vacc] or hatched [Unvacc], respectively) and female (dark [adult] and light [aged] pink, solid [Vacc] or hatched [Unvacc], respectively) mice were challenged with a high dose of ma2009 drift variant virus at 42 days post vaccination. Morbidity was evaluated as the percent change in body mass 3 and 5 days post challenge (n=5-8/group) (a and b). Lung virus titers were measured on days 3 and 5 post challenge (n=5-8/group) (c and d). Data represent means ± standard error of the mean from two independent replications and significant differences between groups are denoted by asterisks (**p*< 0.05) based on two way ANOVAs. Young adult vaccinated males are represented as Vacc. AdM; young adult vaccinated females as Vacc.AdF; aged vaccinated males as Vacc.AgM; and aged vaccinated females as Vacc.AgF. Young adult unvaccinated males are represented as Unvacc. AdM; young adult unvaccinated females as Unvacc. AdF; aged unvaccinated males as Unvacc. AgM; and aged unvaccinated females as Unvacc. AgF.


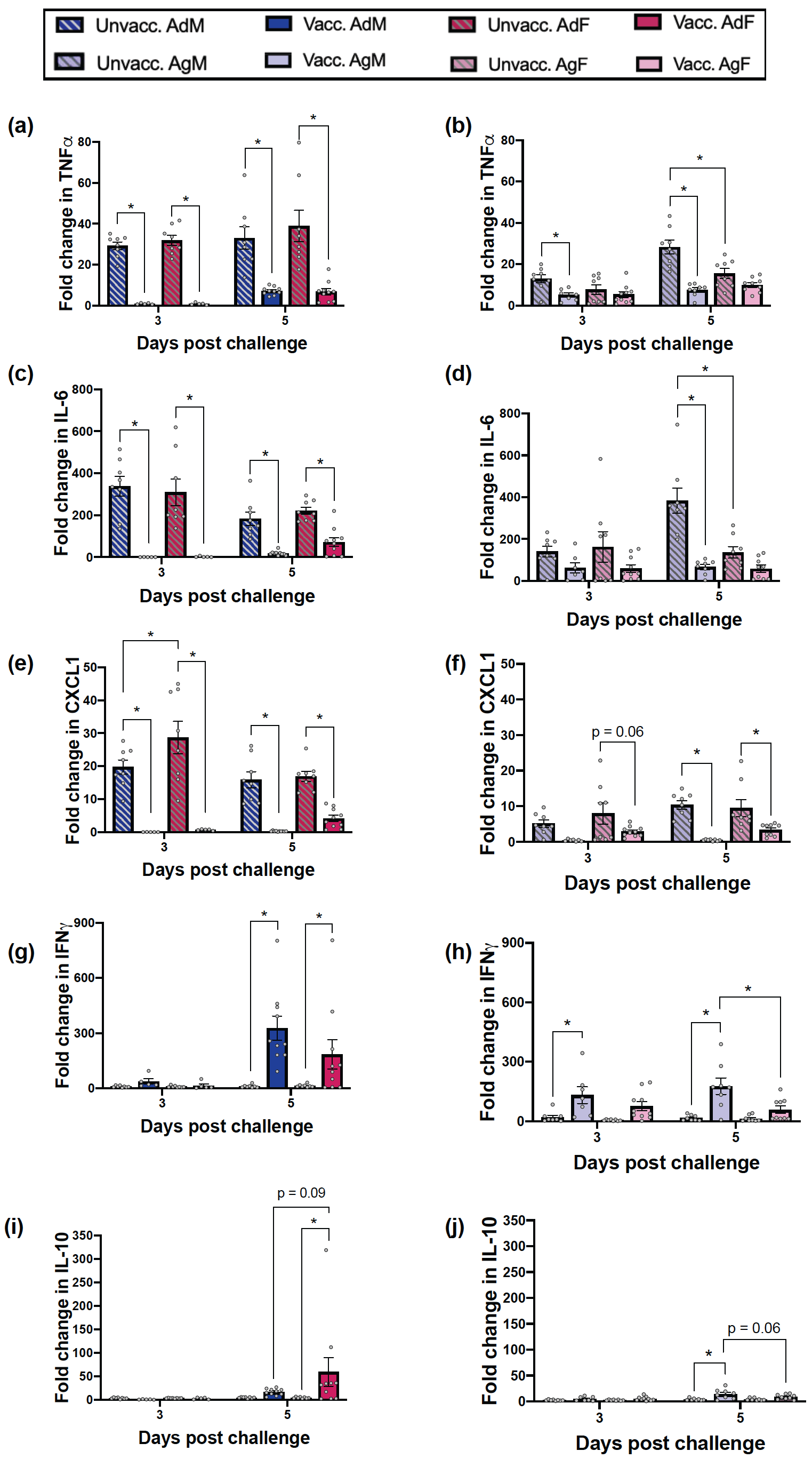


**Supplementary Figure 2: Unvaccinated mice have greater induction of acute phase proteins and lower induction of T-cell cytokines than vaccinated mice, regardless of age or sex.** Vaccinated (Vacc) and unvaccinated (Unvacc) adult (8-10 weeks) and aged (68-70 weeks) male (dark [adult] and light [aged] blue, solid [Vacc] or hatched [Unvacc], respectively) and female (dark [adult] and light [aged] pink, solid [Vacc] or hatched [Unvacc], respectively)mice were challenged with a high dose of ma2009 drift variant virus at 42 days post vaccination. The concentrations of proinflammatory cytokines were assessed prior to and 3 and 5 days post challenge, and the induction (i.e., fold change) from baseline of TNFα (a and b), IL-6 (c and d), CXCL1 (e and f), IFNγ (g and h), and IL-10 (I and j) are presented. Data represents means ± standard error of the mean from two independent replications, and significant differences between groups are denoted by asterisks (**p*< 0.05) based on two way ANOVAs. Young adult vaccinated males are represented as Vacc. AdM; young adult vaccinated females as Vacc.AdF; aged vaccinated males as Vacc.AgM; and aged vaccinated females as Vacc.AgF. Young adult unvaccinated males are represented as Unvacc. AdM; young adult unvaccinated females as Unvacc. AdF; aged unvaccinated males as Unvacc. AgM; and aged unvaccinated females as Unvacc. AgF.
